# Atomic layer deposited films of Al_2_O_3_ on fluorine-doped tin oxide electrodes: stability and barrier properties

**DOI:** 10.3762/bjnano.12.2

**Published:** 2021-01-05

**Authors:** Hana Krýsová, Michael Neumann-Spallart, Hana Tarábková, Pavel Janda, Ladislav Kavan, Josef Krýsa

**Affiliations:** 1 J. Heyrovsky Institute of Physical Chemistry, Czech Academy of Sciences, Dolejškova 2155/3, 182 23 Prague 8, Czech Republic; 2 Department of Inorganic Technology, University of Chemistry and Technology Prague, Technická 5, 166 28 Prague 6, Czech Republic

**Keywords:** Al_2_O_3_, atomic layer deposition (ALD), barrier properties, corrosion, electrochemistry, FTO

## Abstract

Al_2_O_3_ layers were deposited onto electrodes by atomic layer deposition. Solubility and electron-transport blocking were tested. Films deposited onto fluorine-doped tin oxide (FTO, F:SnO_2_/glass) substrates blocked electron transfer to redox couples (ferricyanide/ferrocyanide) in aqueous media. However, these films were rapidly dissolved in 1 M NaOH (≈100 nm/h). The dissolution was slower in 1 M H_2_SO_4_ (1 nm/h) but after 24 h the blocking behaviour was entirely lost. The optimal stability was reached at pH 7.2 where no changes were found up to 24 h and even after 168 h of exposure the changes in the blocking behaviour were still minimal. This behaviour was also observed for protection against direct reduction of FTO.

## Introduction

Surface coverage by thin films for the improvement of mechanical, optical, and electrochemical properties of solid surfaces is of great technological importance. In this context, corrosion, which is an electrochemical process, is the main concern. In an oxidizing and humid atmosphere, disintegration of metallic structural elements into oxides is a process that leads to ultimate loss of these structural elements if they are not properly protected. Turning iron and steel into rust is quantitatively the largest loss factor for structures, vehicles, and machinery worldwide. In some cases, oxides are of technological interest; however, they themselves are subject to deterioration if exposed to extreme basic or acidic conditions and/or to electrochemical reduction. Coating with an extra electrochemically resistant, sufficiently contiguous, and poreless oxide layer may aid in this case. This is the topic of the present work, in which atomic layer deposition (ALD) is used as the coating technique [1]. This method is a gas-phase process which relies on a molecular approach. Therefore, a conformal coating, which reaches the pores and crevasses of the sample, can be obtained.

Protective coating of electrified interfaces is particularly challenging, because electron or hole transport through the coating must be maintained. Previously, ALD and other coating techniques have been shown to protect a semiconducting hematite electrode against corrosion and photocorrosion by using titanium dioxide [2–4]. Depending on the thickness of the protecting layer, the passage of electrical current was progressively hindered as the layer thickness was increased, such that tunnelling became impossible [5]. A similar protection by ALD-grown layers of Ta_2_O_5_ [6] or SiO_2_ [7] was used for other semiconducting electrodes, such as ZnO.

Aluminium oxide is another promising candidate for this task. It is amphoteric but insoluble in aqueous media at a neutral pH value [8–9]. ALD oxide layers, including Al_2_O_3_, were used as barrier coatings on copper to protect against corrosion in 0.1 M NaCl [10]. As-deposited ALD Al_2_O_3_ films are typically amorphous with a poor resistance to chemical attack [11–13]. These films do not withstand, for example, exposition to environmental media, such as 5% NaCl and sea water, to diluted HCl and H_2_SO_4_ (pH 4) [11], to acidic (1 M H_2_SO_4_) or alkaline (1 M NaOH) solutions [12], or to solutions consisting of 6% NH_4_OH and 5% H_2_O_2_ at 80 °C or 1% HF at 21 °C, employed in the manufacturing of electronics [13]. ALD Al_2_O_3_ treated at approx. 900 °C exhibits a significant improvement regarding chemical stability, which is explained by the densification and transition of amorphous to polycrystalline γ-Al_2_O_3_ [12] or to oriented θ-Al_2_O_3_ [13]. However, on thermodynamic grounds, alumina is soluble in both acidic and alkaline media [8]. Besides protection, Al_2_O_3_ ALD layers have also been used for passivating surface states on water-oxidizing hematite photoanodes [14–15].

Very thin layers of insulators may allow for electron transport across these layers if tunnelling occurs. It will be shown in the next sections that, for the thinnest deposited layers, this process is responsible for electrical currents passing across bulk solid/Al_2_O_3_/liquid interfaces. A special feature of alumina coatings was ascribed to its capability of passivating semiconductor/electrolyte interfaces, thus reducing photogenerated charge-carrier recombination (e.g., on BiVO_4_ [16]).

In this work, Al_2_O_3_ films were deposited via ALD on thermally grown SiO_2_ on silicon or on fluorine-doped tin oxide (FTO, F:SnO_2_ on glass). The resistance to dissolution in aqueous solutions of various pH values was tested as well as the blocking capability of electron transport as criteria for the presence or change of Al_2_O_3_ on the surface.

## Experimental

K_3_[Fe(CN)_6_], K_4_[Fe(CN)_6_], KCl, NaOH, and H_2_SO_4_ were of analytical grade. 0.1 M sodium phosphate buffer solution, pH 7.2, was obtained from Fluka. Triply distilled water was used for the preparation of solutions.

Fluorine-doped tin oxide-coated 2 mm thick glass (TEC-7, 7 Ω/□, Sigma-Aldrich), was ultrasonically pre-cleaned with isopropanol, treated with acetone, ethanol, and water and dried in a stream of argon. Si(100) wafers with a 300 nm thick thermal oxide layer (Silicon Quest International, USA) were treated successively with acetone/ethanol/water.

Al_2_O_3_ films were grown by using an ALD system R200 (Picosun, Finland) in the thermal mode with varying numbers of identical deposition cycles. Trimethylaluminium (TMA) and water (both from Strem Chemicals, Inc.) were used as precursors. The temperature range for all the organometallic aluminium precursors was 30–300 °C [17]. The deposition of Al_2_O_3_ was performed at 300 °C, as recommended [4]. The pulse of the TMA precursor was 0.1 s with a purge time of 5 s and the pulse of H_2_O was 0.1 s with a purge time of 10 s. TMA and water were maintained at 22 °C. Nitrogen (99.999%, Linde) was used as a carrier gas. The deposition of aluminium oxide films on silicon or FTO was carried out by performing 30, 60, 120, and 200 cycles.

The layer thickness of the films was determined via referenced spectroscopic ellipsometry (RSE, Accurion). In the range of 10 to 200 deposition cycles, the measured deposition rate was 0.085 nm/cycle, corresponding to layer thickness values of 2.5, 5, 10, and 17 nm, respectively.

For dissolution studies, Al_2_O_3_ films on FTO were exposed, at various time intervals and at room temperature, to 1 M NaOH, 1 M H_2_SO_4_, and buffered solution (pH 7.2).

Electrochemical experiments were carried out in a single-compartment three-electrode cell using a Zahner workstation. The reference electrode was Ag/AgCl (3 M KCl) and a platinum rod was used as the counter electrode. The blocking properties of the deposited layers were evaluated by cyclic voltammetry (CV) in an aqueous electrolyte composed of 0.5 mM K_3_[Fe(CN)_6_] and 0.5 mM K_4_[Fe(CN)_6_] in 0.5 M KCl (pH 2.5, adjusted with HCl) or in phosphate buffer (pH 7.2). Chronoamperometric measurements were performed in buffered solution (pH 7.2) at −1.2 V (vs Ag/AgCl).

The morphology of the films was characterized ex situ, under ambient conditions, by atomic force microscopy (AFM, Dimension Icon, Bruker, USA) in a semicontact (tapping) mode. A silicon cantilever (TESPA-V2) with a resonant frequency *f*_res_ of approx. 300 kHz, a spring constant *k* of 0.42 N·m^−1^, and a nominal tip radius of 8 nm (Bruker, USA) was employed. The Gwyddion software (v. 2.53) was utilized for processing AFM image data.

## Results and Discussion

**Figure 1:**
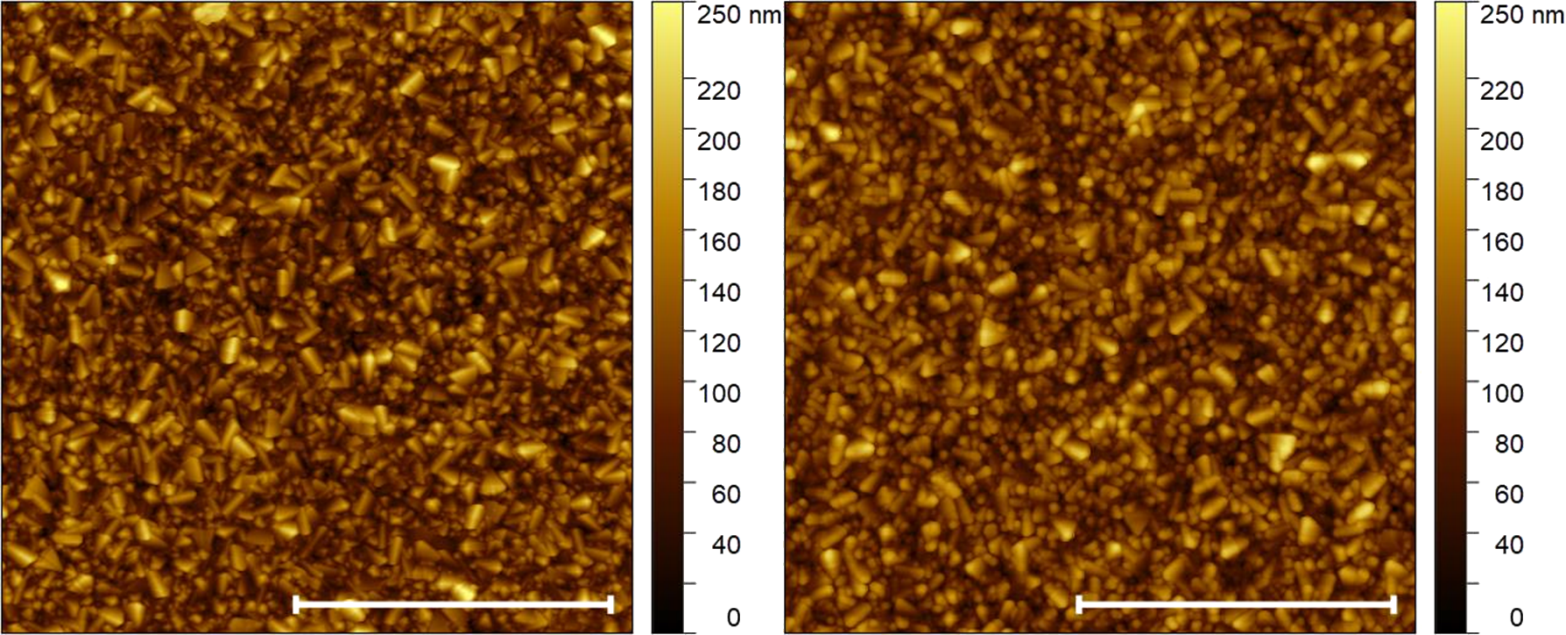
AFM topography image (10 µm × 10 µm) of an FTO substrate (left) and of an FTO substrate coated with a 17 nm thick Al_2_O_3_ layer (right). White bars represent 5 µm.

AFM was used to compare the morphology of the substrates before and after ALD deposition of an Al_2_O_3_ layer. As shown in Figure S1 and Figure S2 (Supporting Information File 1), the surface morphology of both substrates, the as-received and the Al_2_O_3_-coated SiO_2_ layer, was almost identical. This indicates a uniform distribution of the deposited Al_2_O_3_. The height-density distribution (Figure S2, Supporting Information File 1), calculated from the AFM topography images (Figure 1), shows that the deposition of Al_2_O_3_ onto FTO substrates does not change its surface morphology.

Calculated RMS (root mean square) values for AFM images of FTO and FTO coated with a 17 nm Al_2_O_3_ layer were practically the same (i.e., 35 and 34 nm, respectively). This shows that Al_2_O_3_ was conformally deposited onto the FTO surface.

Next, the blocking properties of the aluminium oxide layers were tested via cyclic voltammetry on ALD-deposited Al_2_O_3_ films of various thickness on FTO substrates in an electrolyte containing 0.5 mM K_3_[Fe(CN)_6_] and 0.5 mM K_4_[Fe(CN)_6_] in 0.5 M KCl. This redox couple produces a reversible wave in CV. The magnitude of the voltammetric current was taken as an indicator of the blocking quality of the coating layer: the lower the value, the better the blocking. This approach has been used previously [18–20] for testing semiconducting nonporous blocking layers of oxides (TiO_2_ or SnO_2_) deposited onto FTO. In this way, direct electron transfer between the redox couple in the electrolyte solution and the conducting substrate (i.e., FTO), at sites that were not covered by the semiconductor, was blocked. This blocking layer (also called electron-selective layer) is a key component of dye-sensitized [19] and perovskite solar cells [21]. The blocking function consists in supporting vectorial electron transport from a photoexcited light absorber (sensitizing dye or perovskite) to the negative terminal of the solar cell, usually an FTO or a similar transparent conducting oxide. At the same time, this layer blocks the back electron transfer from the current collector (FTO) to the electrolyte redox mediator, to the hole-transporting medium, or to the perovskite (depending on the device type). This parasitic effect occurs through defects, such as pinholes and cracks in the blocking layer. Their presence is identified by the occurrence of anodic currents assigned to the oxidation of [Fe(CN)_6_]^4−^ at FTO areas exposed by these defect sites [18]. Promising properties of Al_2_O_3_ blocking layers for dye-sensitized solar cells were first reported by Palomares at al. [22].

**Figure 2:**
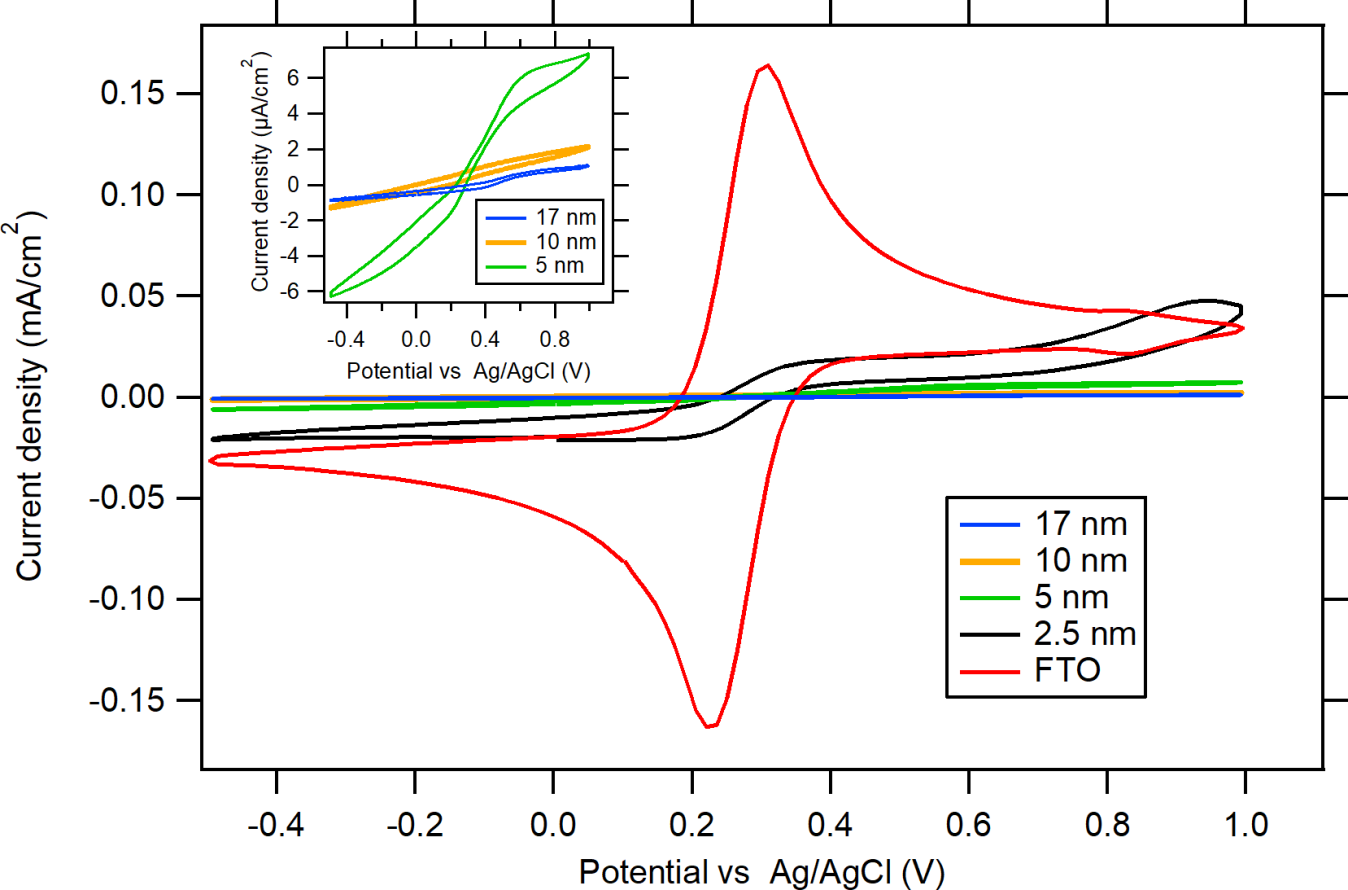
CVs of 0.5 mM K_3_[Fe(CN)_6_] and 0.5 mM K_4_[Fe(CN)_6_] in 0.5 M KCl on FTO electrodes covered with Al_2_O_3_ films with thickness values of 2.5, 5, 10 and 17 nm, respectively. Inset: details of 5, 10, and 17 nm thick alumina-coated samples. The scan rate is 50 mV/s.

The CVs in Figure 2 demonstrate the blocking behaviour of Al_2_O_3_ films on FTO. Increasing numbers of ALD cycles led to an increasing suppression of the peak heights in the CVs. Aluminium oxide films of 10 and 17 nm (corresponding to 120 and 200 ALD cycles, respectively) were almost completely blocked (Figure 2).

The evaluation of the blocking behaviour of FTO by the aluminium oxide layer is based on the measurement of the voltammetric peak current density, *j*_p_, which follows the Randless–Ševčík equation, and on the calculation of the effective pinhole area (EPA), as described in detail in our previous work [18,20] and in Supporting Information File 1. For all the voltammograms of Al_2_O_3_ films on FTO (Figure 2), the voltammetric peak separation (Δ*E*_pp_) normalized to that of pure FTO is higher than three. This means that there are B-type defects in the barrier film. These defects cause not only the delamination of the Al_2_O_3_ film from the FTO substrate, but also a slowdown of the charge-transfer kinetics (accompanied by a strong increase in Δ*E*_pp_).

The Table 1 shows the difference between the blocking properties of Al_2_O_3_ layers of various thickness values. The effective pinhole area gradualy decreases with an increase in the thickness values of the Al_2_O_3_ layer, reaching 0.24% for a 17 nm thick film.

**Table 1:** Analysis of EPA and of the type of defect for as-deposited Al_2_O_3_ films of various thickness values. Data from cyclic voltammetry of [Fe(CN)_6_]^3−/4−^, shown in Figure 2 and in Figure S4, Supporting Information File 1.

	*j*_p_ (μA cm^−2^)^a^	EPA (%)	Defect type

FTO	164 (0.3 V)	—	—
FTO + 2.5 nm Al_2_O_3_	19.8 (0.5 V)	12.1	B
FTO + 5 nm Al_2_O_3_	6.38 (0.65 V)	3.89	B
FTO + 10 nm Al_2_O_3_	1.26 (0.5 V)	0.77	B
FTO + 17 nm Al_2_O_3_	0.393 (0.5 V)	0.24	B
…………………………………………………………………………………..			
FTO + 17 nm Al_2_O_3_ (5 min in 1 M NaOH)	3.70 (0.5 V)	2.28	B

^a^*j*_p_ values were determined for the electrode potential vs Ag/AgCl (in parenthesis).

### Exposure to NaOH

**Figure 3:**
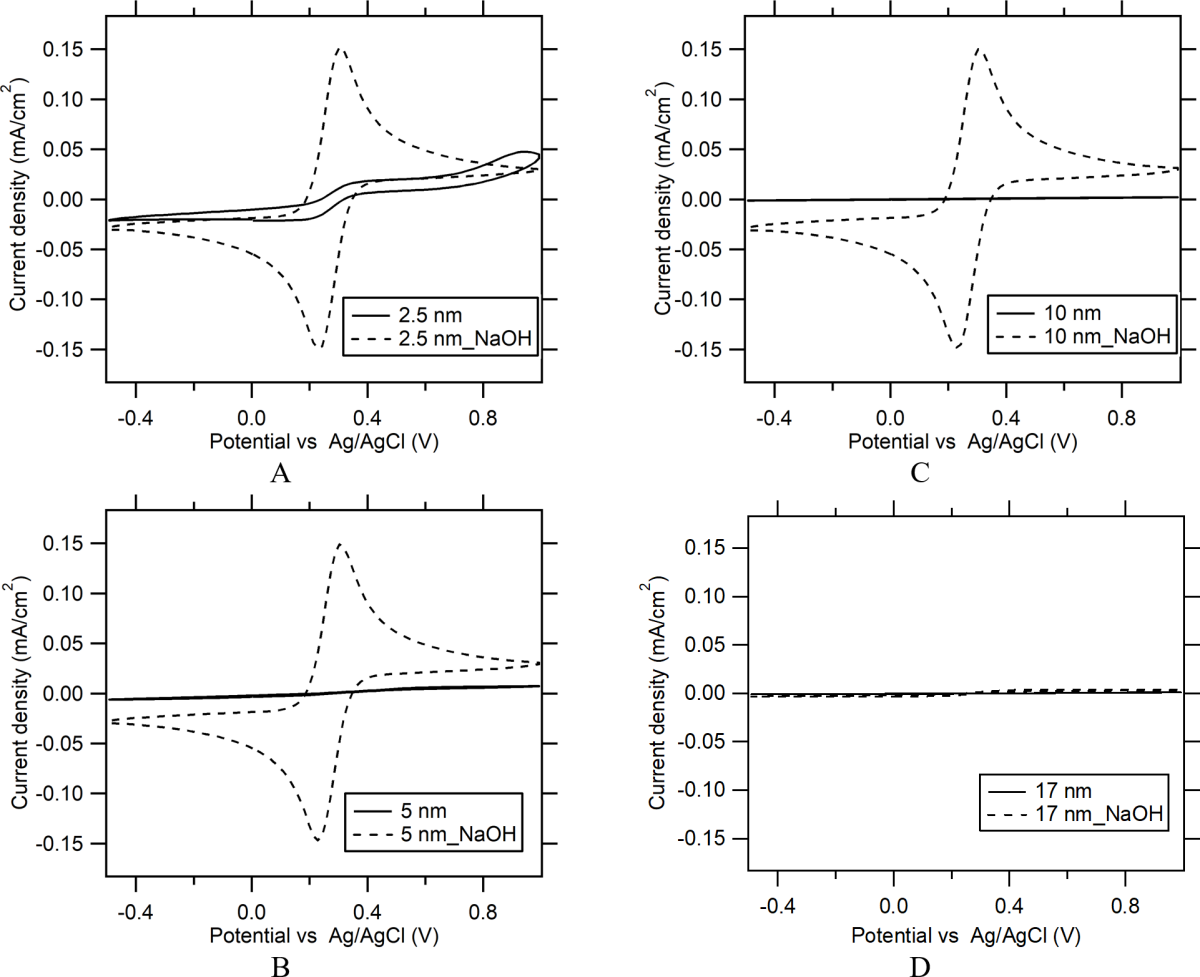
CVs of 0.5 mM K_3_[Fe(CN)_6_] and 0.5 mM K_4_[Fe(CN)_6_] in 0.5 M KCl demonstrating the blocking properties of Al_2_O_3_ films. Al_2_O_3_ films of thickness values of (A) 2.5, (B) 5, (C) 10, and (D) 17 nm, respectively, before and after exposure to 1 M NaOH for 5 min. The scan rate is 50 mV/s.

Al_2_O_3_ films, of thickness values of 2.5, 5, 10 and 17 nm, deposited onto FTO substrates were immersed in a 1 M NaOH solution, for 5 min at room temperature. The dissolution of the Al_2_O_3_ films was fast and only the thickest (17 nm) Al_2_O_3_ film was not completely dissolved (the FTO substrate was still covered) (Figure 3).

**Figure 4:**
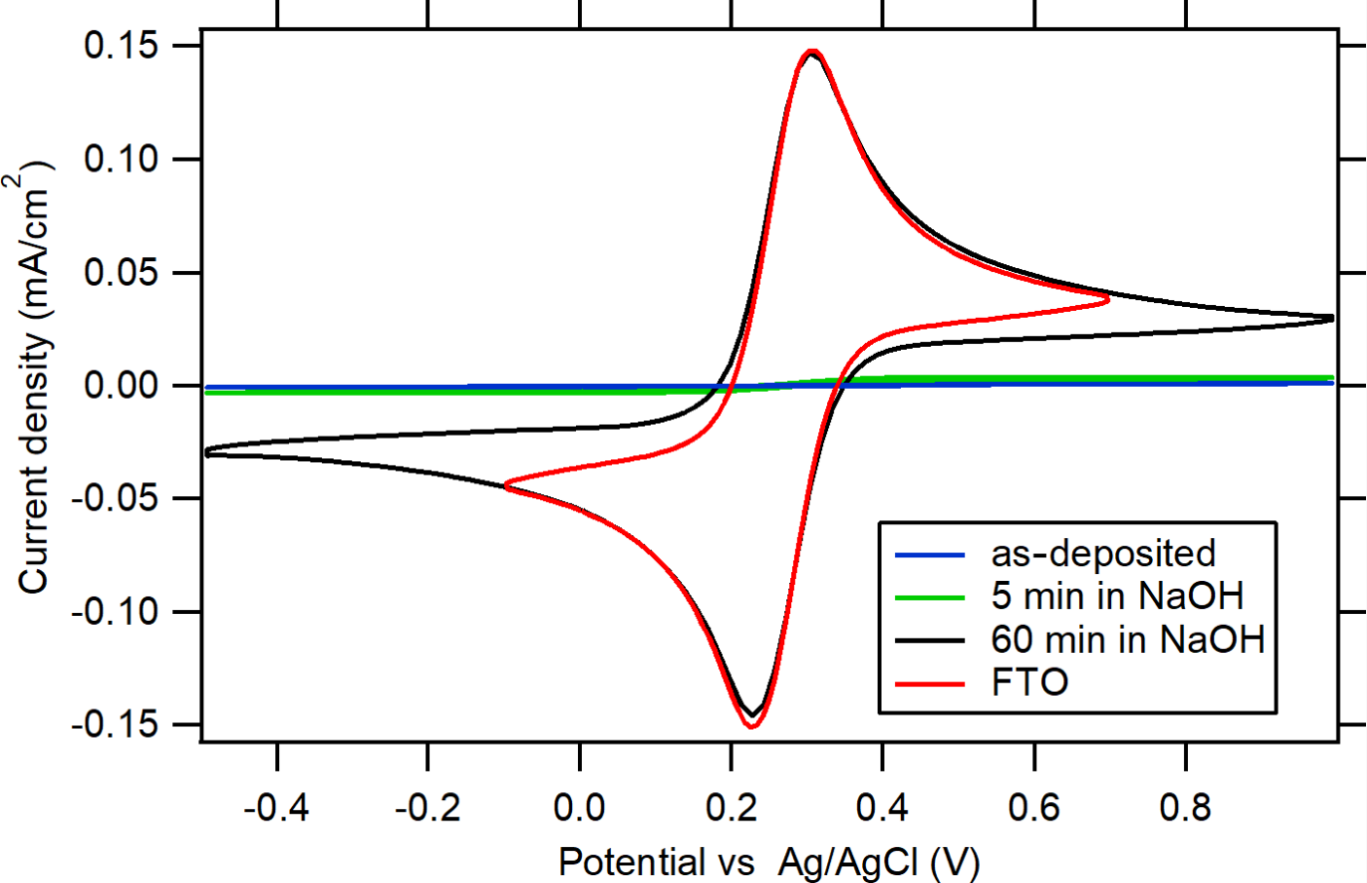
CVs of 0.5 mM K_3_[Fe(CN)_6_] and 0.5 mM K_4_[Fe(CN)_6_] in 0.5 M KCl demonstrating the blocking properties of 17 nm thick Al_2_O_3_ films on FTO before and after exposure to 1 M NaOH for 5 or 60 min compared to uncovered FTO. The scan rate is 50 mV/s.

The electrochemical response was obtained as a function of the exposure time (Figure 4). It can be implied that, even after a short exposure to a 1 M NaOH solution (5 min), a significant part of the Al_2_O_3_ layer remained on the substrate. A longer exposure time (60 min) resulted in a complete dissolution of the ALD film, and the voltammogram of the sample resembled that of pure FTO, indicating that a significant charge transfer occurred at the electrolyte solution/FTO interface. The blocking properties of a 17 nm thick Al_2_O_3_ film after a 5 min exposure to 1 M NaOH were evaluated via EPA calculation and shown in Table 1. A 5 min exposure to 1 M NaOH resulted in a decrease of the blocking properties of the Al_2_O_3_ films (increase of EPA from 0.24% to 2.28%). This value of EPA is between the EPA values for 5 and 10 nm thick Al_2_O_3_ films (Figure S4, Supporting Information File 1). Based on the EPA extrapolation between the thickness values of 5 and 10 nm, the remaining thickness of the Al_2_O_3_ film on FTO was in the range of 7–8 nm. This suggests that during the 5 min exposure to 1 M NaOH, approx. 9–10 nm of the ALD film was dissolved, which corresponds to a dissolution rate of approx. 108–120 nm/h.

**Figure 5:**
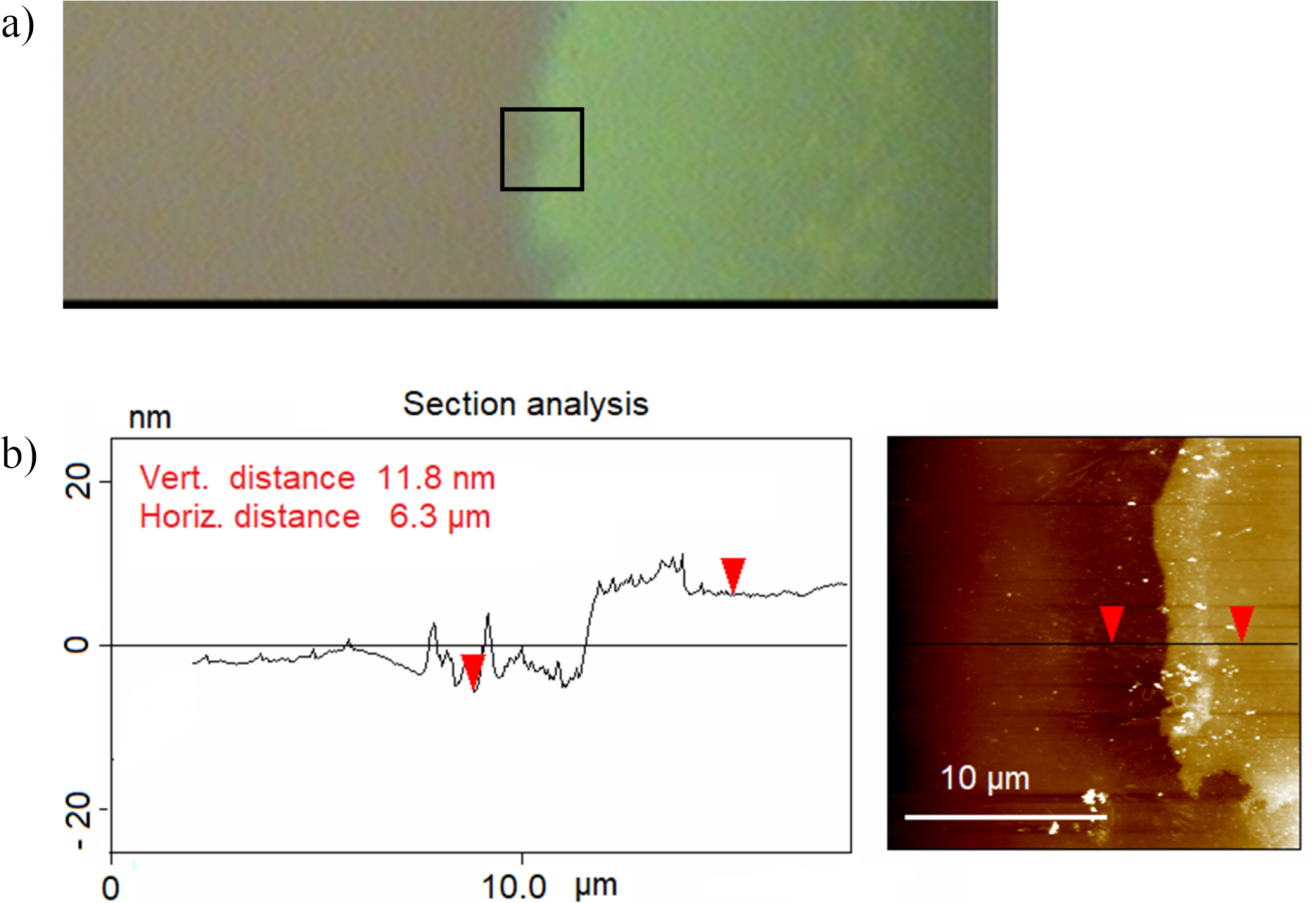
Si wafer coated with an Al_2_O_3_ film (17 nm) after exposure to 1 M NaOH for 1h. (a) Optical microscopy image of the border between an unexposed (green, right side) and an exposed (grey/violet, left side) area of the Al_2_O_3_ film. The black square shows an area of 20 µm × 20 µm, where the AFM measurement shown in (b) was carried out. (b) AFM image and line analysis.

When a Si/SiO_2_ wafer coated with Al_2_O_3_ (17 nm) was exposed to a drop (10 μL) of 1 M NaOH for 1 h, the AFM data showed a height change between the unexposed and the exposed areas of the Al_2_O_3_ film. During this exposure, the increase in the drop size was negligible. The unexposed Al_2_O_3_ film (Figure 5a, dark green area) appears to be compact. The AFM line analysis (Figure 5b) shows a height difference of 12 nm between the area exposed for 1 h and the unexposed area. The electrochemical analysis of blocking behaviour showed that Al_2_O_3_ films were completely dissolved after 1 h of exposure. Therefore, the effective thickness of the film was approx. 12 nm. This result is comparable to the thickness value of 17 nm measured via RSE.

### Exposure to H_2_SO_4_

**Figure 6:**
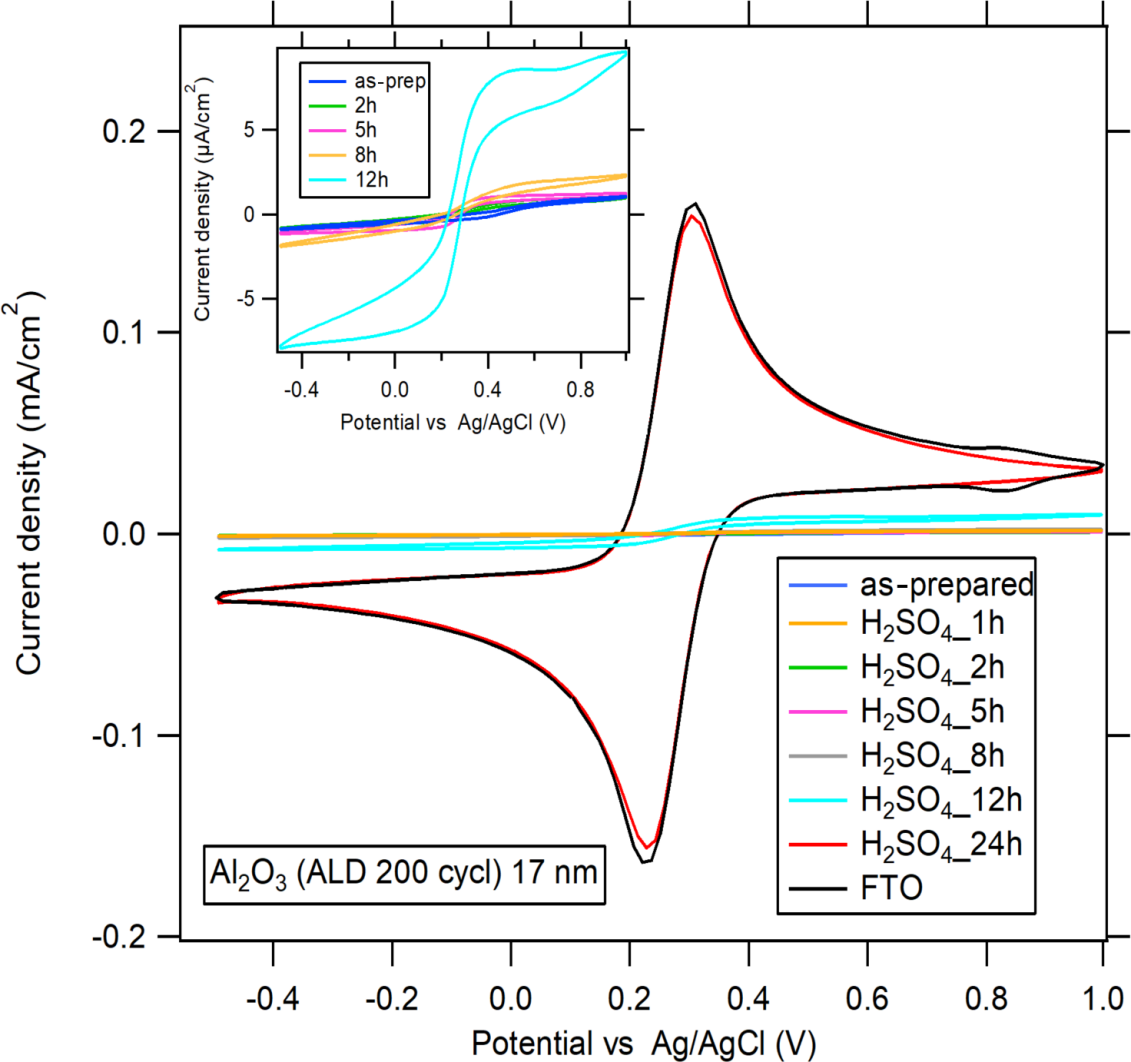
CVs of 0.5 mM K_3_[Fe(CN)_6_] and 0.5 mM K_4_[Fe(CN)_6_] in 0.5 M KCl demonstrating blocking properties of 17 nm thick Al_2_O_3_ films before and after exposure to 1 M H_2_SO_4_ for 1, 2, 5, 8, 12, and 24 h compared to uncoated FTO. Inset: CVs for the first 12 h. The scan rate is 50 mV/s.

The results for the exposure to sulfuric acid (Figure 6) are similar to those obtained for the exposure to alkaline solutions: after 24 h in sulfuric acid, also 17 nm thick Al_2_O_3_ layers were dissolved as the CV curves resembled those of FTO. However, the decomposition of Al_2_O_3_ films in sulfuric acid was much slower than in NaOH, since after a 12 h exposure the blocking properties were still very good and resembled those of unexposed 5 nm thick ALD layers (Figure 2). From this, a dissolution rate of approx. 1 nm/h was estimated.

The voltammogram of bare FTO (Figure 6, black curve) exibited additional waves near 0.8–0.9 V assigned to a Prussian blue deposit, which is known to sometimes interfere with the blocking tests with ferrocyanide/ferricyanide [23]. Interestingly, a clean surface (free of Prussian blue) was observed when the Al_2_O_3_-protected electrode was denuded by the dissolution of its coating, both in acidic (Figure 6) and alkaline (Figure 4) media.

### Exposure to phosphate buffer (pH 7.2)

**Figure 7:**
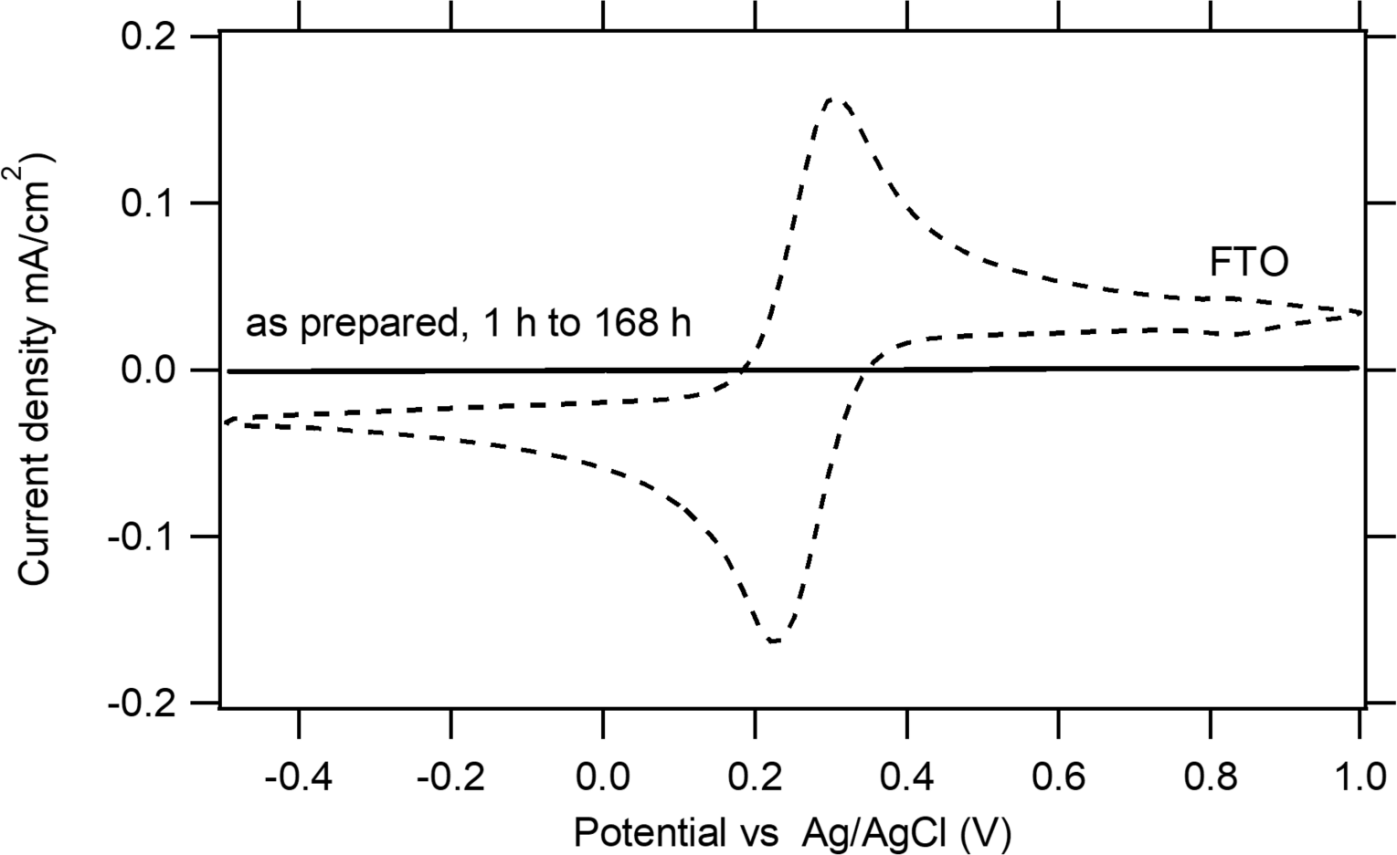
CVs in the presence of 0.5 mM K_3_[Fe(CN)_6_] and 0.5 mM K_4_[Fe(CN)_6_] in 0.5 M KCl. Blocking properties of samples coated with 17 nm thick Al_2_O_3_ films before and after exposure to phosphate buffer (pH 7.2) for 1, 2, 24, 48, and 168 h, compared to bare FTO. The scan rate is 50 mV/s.

Cyclic voltammetry curves of 17 nm thick Al_2_O_3_ films on FTO substrates (in the presence of 0.5 mM K_3_[Fe(CN)_6_] and 0.5 mM K_4_[Fe(CN)_6_]) exposed to phosphate buffer are shown in Figure 7. The blocking properties of Al_2_O_3_ films upon exposure to phosphate buffer (pH 7.2) were very good even after a prolonged exposure (1 week). Figure S5 (Supporting Information File 1) shows that the blocking properties of Al_2_O_3_ films remained almost unchanged after one week of exposure. The observed stability in solutions of neutral pH is in agreement with published dissolution data, which show a minimum solubility at pH 6 [8].

Another test to assess the blocking properties of ALD Al_2_O_3_ films consisted in exploring the electrochemical reduction of the FTO film itself resulting in metallic tin, as evidenced by the dark coloration of the samples after a prolonged polarization at −1.2 V, according to Equation 1:

[1] $${\text{SnO}}_{2}+4{\text{H}}^{+}+4e^{-}\to \text{Sn}+2{\text{H}}_{2}\text{O}$$

**Figure 8:**
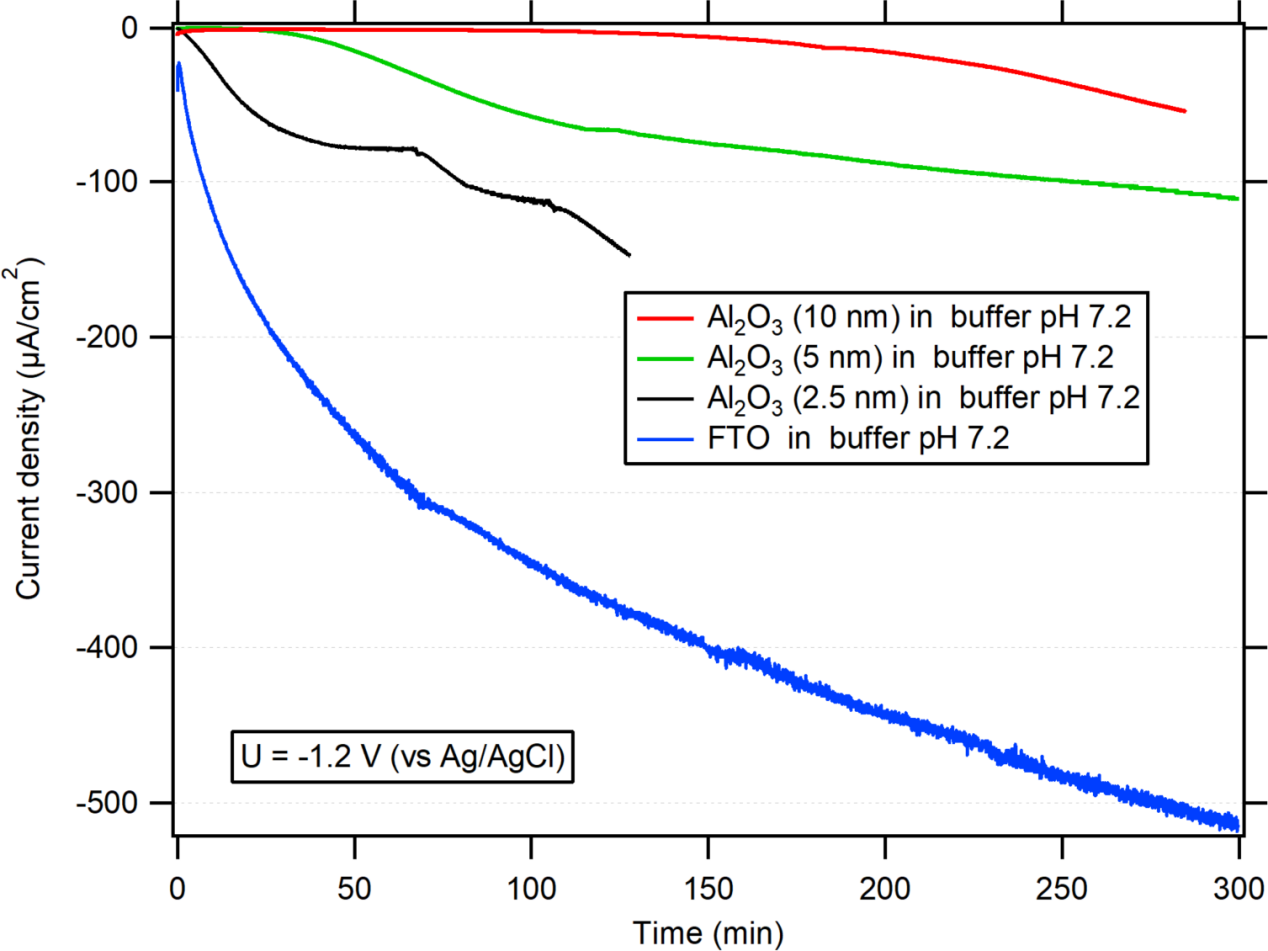
Chronoamperometry in buffered solution (pH 7.2). Comparison of bare FTO (blue) and FTO covered with a 10 nm Al_2_O_3_ layer (red), a 5 nm Al_2_O_3_ layer (green), or with a 2.5 nm Al_2_O_3_ layer (black). The applied potential was −1.2 V vs Ag/AgCl. The solution was stirred and Ar-bubbled.

The AFM phase images of glass/FTO before and after electrochemical treatment (−1.2 V, 5 h, phosphate buffer, pH 7.2) are shown in Figure S6 (Supporting Information File 1). The change in the surface nanoscale morphology can be explained by the replacement of SnO_2_ by Sn. Figure 8 and Table 2 show that increasing thickness values of ALD alumina layers led to an increase in the efficacy of suppression of the reduction of the FTO film. A thickness of 10 nm was sufficient to protect the FTO layer. This finding was also supported by AFM phase images of a glass/FTO/10 nm Al_2_O_3_ film after the electrochemical treatment (Figure S7, Supporting Information File 1). While bare FTO shows a morphological change on its surface (Figure S6, Supporting Information File 1), the surface of an FTO substrate covered by a thin conformal Al_2_O_3_ film does not show any significant morphological changes after the electrochemical treatment (Figure S7, Supporting Information File 1).

**Table 2:** Photographs of bare FTO substrates and of Al_2_O_3_ layers on FTO substrates after polarization at −1.2 V vs Ag/AgCl, in buffer (pH 7.2) at different time intervals.^a^

FTO	FTO	2.5 nm Al_2_O_3_	5 nm Al_2_O_3_	10 nm Al_2_O_3_
1 h	5 h	2 h	5 h	5 h

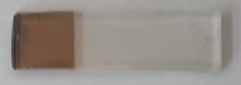	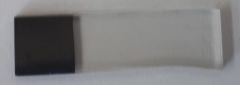	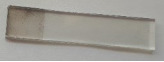	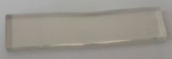	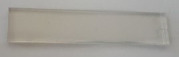

^a^Colored areas in the left part of each sample correspond to areas that received the electrochemical treatment. The right parts of the samples were not immersed in the electrolyte.

The cathodic breakdown of FTO is also identified by significant changes observed in the cyclic voltammogram of the [Fe(CN)_6_]^3−/4−^ couple (Figure S8, Supporting Information File 1). This redox couple showed a nearly reversible behaviour in the voltammogram of bare FTO. However, the irreversibility (quantified by peak-to-peak separation) became larger when the cathodic vertex potential was extended below approx. −1 V. Eventually, the voltammetric waves of [Fe(CN)_6_]^3−/4−^ disappeared when the FTO substrate was destroyed at very negative potentials. We observed complex voltammetric features acompanying the cathodic attack of FTO (Equation 1). Conversely, the Al_2_O_3_-coated FTO substrate was efficiently protected against these effects. The main feature of the protected FTO was a strong cathodic current (of the order of several mA/cm^2^) with an onset potential of approx. −1.7 V. The reduction of ferricyanide only slightly contributed to the cathodic current (on the order of 0.01 mA/cm^2^). Hence, this current is mainly assigned to water reduction. This mechanism is still unclear at this stage of our research.

**Figure 9:**
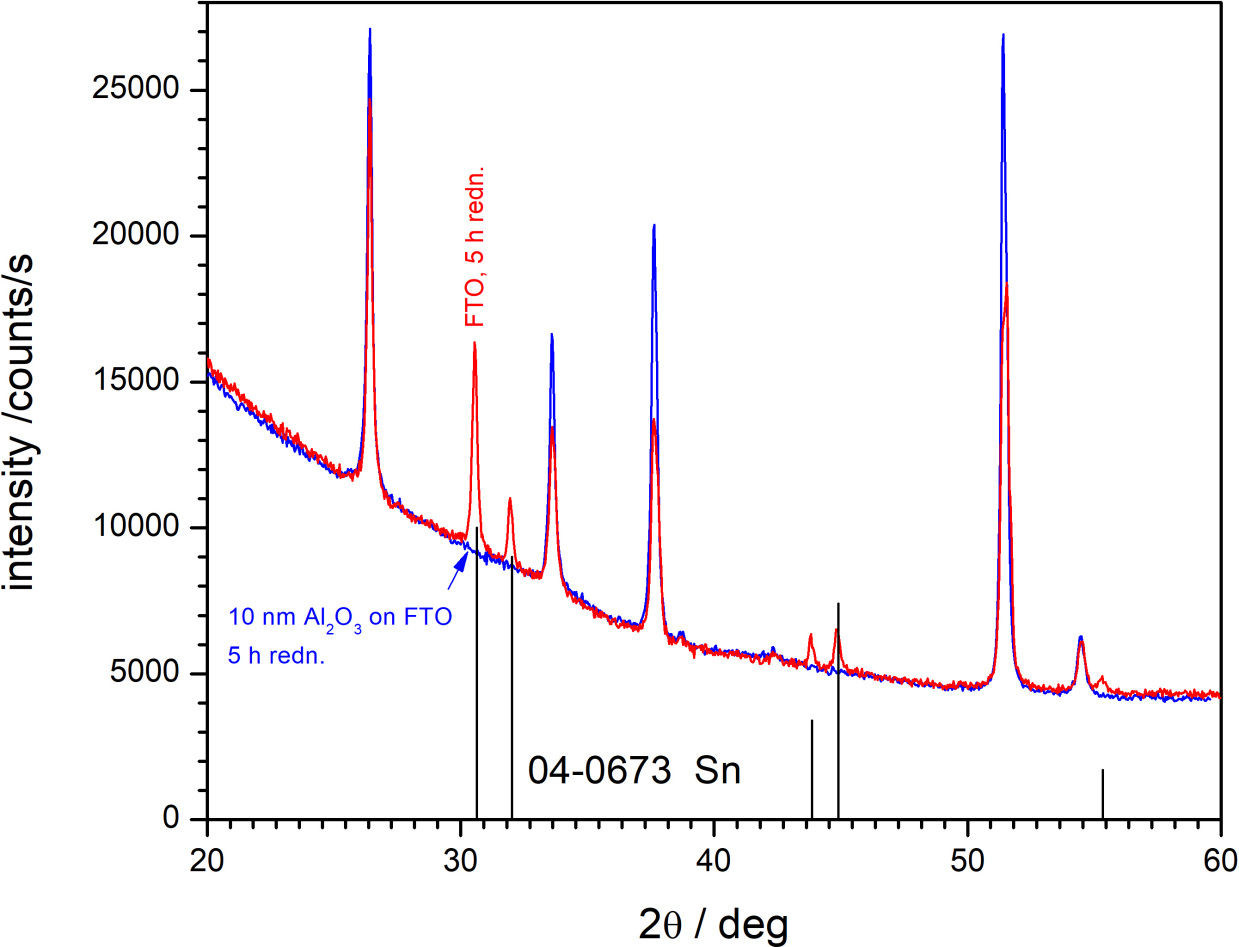
XRD patterns of samples polarized at −1.2 V vs Ag/AgCl in buffer (pH 7.2) for 5 h. Red trace: FTO, blue trace: FTO covered by a 10 nm ALD Al_2_O_3_ film. The Sn pattern was obtained from [24].

X-ray diffraction patterns of unprotected FTO layers, polarized for 1 and 5 h, are shown in Figure S9 (Supporting Information File 1). Upon polarization at −1.2 V vs Ag/AgCl, the reduction of FTO to tin took place (Equation 1). No Sn was found when the FTO layers were protected by alumina films (Figure 9).

## Conclusion

ALD Al_2_O_3_ films, already at a thickness of 5 nm, exhibited very good blocking of the electron transport from solid electrodes (FTO) to a redox couple in aqueous solution.

ALD Al_2_O_3_ films on FTO dissolved rapidly in a 1 M NaOH solution. After 1 h of exposure, a CV curve with the ferricyanide/ferrocyanide couple approached that for uncovered FTO. This corresponded to a dissolution rate of ≈100 nm/h. The films dissolved slower in 1 M H_2_SO_4_. No signs of dissolution of the film were observed after a 17 nm thick alumina layer was exposed for 3 h. Only after 24 h the blocking behaviour was entirely lost. This corresponded to a dissolution rate of 1 nm/h. The films were found to be stable in buffer (pH 7.2) up to 24 h. After 1 week (168 h) of exposure, the changes in the blocking behaviour were minimal. These findings suggest that thin (approx. 5 nm) ALD Al_2_O_3_ films can be used as protecting or passivating overlayers in electrodes, but only when exposed to neutral electrolytes.

The Al_2_O_3_ layers also provided an effective protection against the reduction of FTO. While bare FTO was reduced to Sn at −1.2 V vs Ag/AgCl in a neutral electrolyte, the Al_2_O_3_-coated FTO became reduction-resistant.

## Supporting Information

File 1 Additional figures.
