# Supplementary material for: Atomic layer deposited films of Al2O3 on fluorine-doped tin oxide electrodes: stability and barrier properties
Source: Beilstein J Nanotechnol. 2021 Jan 5;12:24–34. doi: 10.3762/bjnano.12.2 (PMC7801786; doi:10.3762/bjnano.12.2)
Supplement: File 1 — Additional figures. [file Beilstein_J_Nanotechnol-12-24-s001.pdf]

BEILSTEIN JOURNAL OF NANOTECHNOLOGY

Supporting Information

for

Atomic layer deposited films of Al_2O_3 on fluorine-doped tin oxide electrodes: stability and barrier properties

Hana Krýsová, Michael Neumann-Spallart, Hana Tarábková, Pavel Janda, Ladislav Kavan and Josef Krýsa

Beilstein J. Nanotechnol. **2021,** *12,* 24–34. doi:10.3762/bjnano.12.2

Additional figures

License and Terms: This is a supporting information file under the terms of the Creative Commons Attribution License (https://creativecommons.org/licenses/by/4.0). Please note that the reuse, redistribution and reproduction in particular requires that the author(s) and source are credited and that individual graphics may be subject to special legal provisions.
The license is subject to the *Beilstein Journal of Nanotechnology* terms and conditions: (https://www.beilstein-journals.org/bjnano/terms)

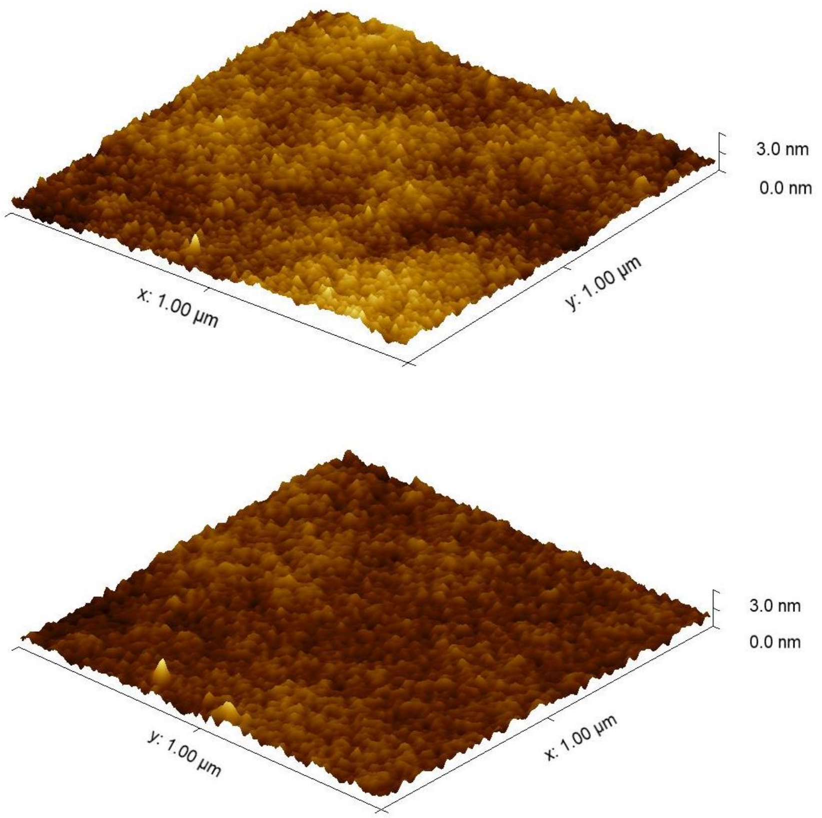

Figure S1: AFM surface morphology of Si/SiO_2 (bottom) and Si/SiO_2 coated with 17 nm Al_2O_3 (top).

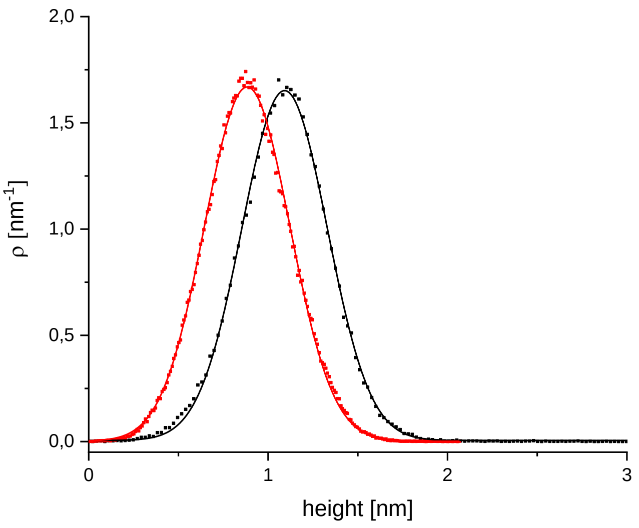

Figure S2: Height-density distribution calculated from AFM topography images (1 µm × 1 µm) of Si/SiO_2 (black dots) and Si/SiO_2 coated with 17 nm Al_2O_3 (red dots).

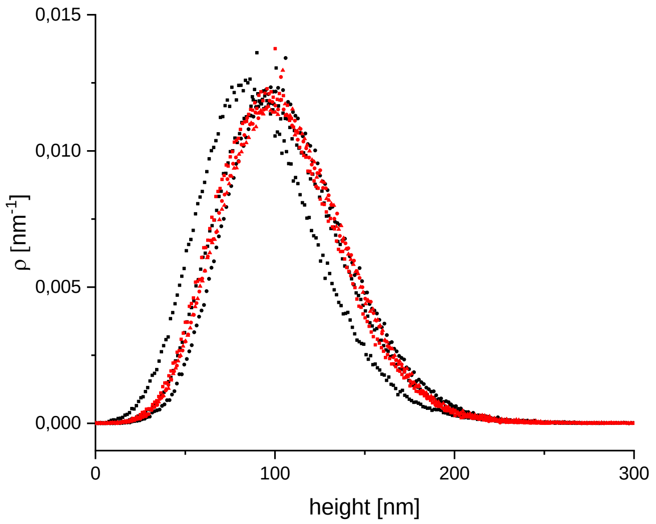

Figure S3: Height-density distribution calculated from AFM topography images (10 µm × 10 µm) of three locations on the FTO substrate (black dots) and on FTO coated by 17 nm Al_2O_3 (red dots).

The effective pinhole area (EPA), defined as a ratio of A_u to A_0, can be expressed as ratio of j_p to $j_{p,FTO}$, where j_p is the peak current density (with reference to the projected electrode area, A_0) measured at the actual blocking electrode. A_u is the uncovered area of FTO and $j_{p,FTO}$ is the current density peak measured at a bare FTO electrode. As suggested in previous works [1,2], there are two types of defects in the barrier film: i) The "defect A", in which the partially blocked electrode behaves like "clean" FTO but with a relatively smaller effective area. The relative increase of the voltammetric peak separation (ΔE_{pp}) on the blocking layer normalized to that of pure FTO is smaller than three (ΔE_{pp} is defined as the difference between the peak potential values for the $Fe(CN)_6{}^{4-}$ oxidation and $Fe(CN)_6{}^{3-}$ reduction). ii) The "defect B" is a more complex situation, in which the defect not only causes the delamination of the titania film from the FTO substrate, but also the slowdown of charge transfer kinetics (accompanied by a strong increase in ΔE_{pp}).

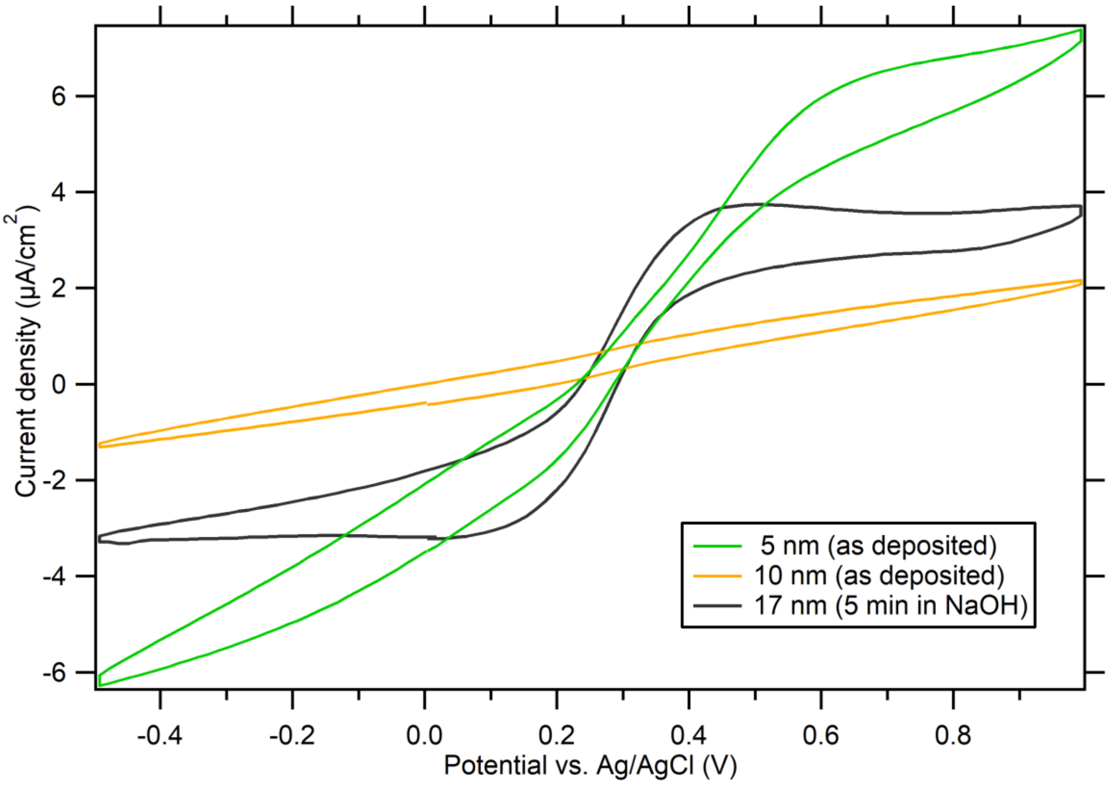

Figure S4: CVs of 0.5 mM $K_3[Fe(CN)_6]$ and 0.5 mM $K_4[Fe(CN)_6]$ in 0.5 M KCl, demonstrating the blocking properties of 5 and 10 nm Al_2O_3 films on FTO electrodes compared to 17 nm Al_2O_3 films on FTO, after an exposure to 1 M NaOH for 5 min. The scan rate is 50 mV/s.

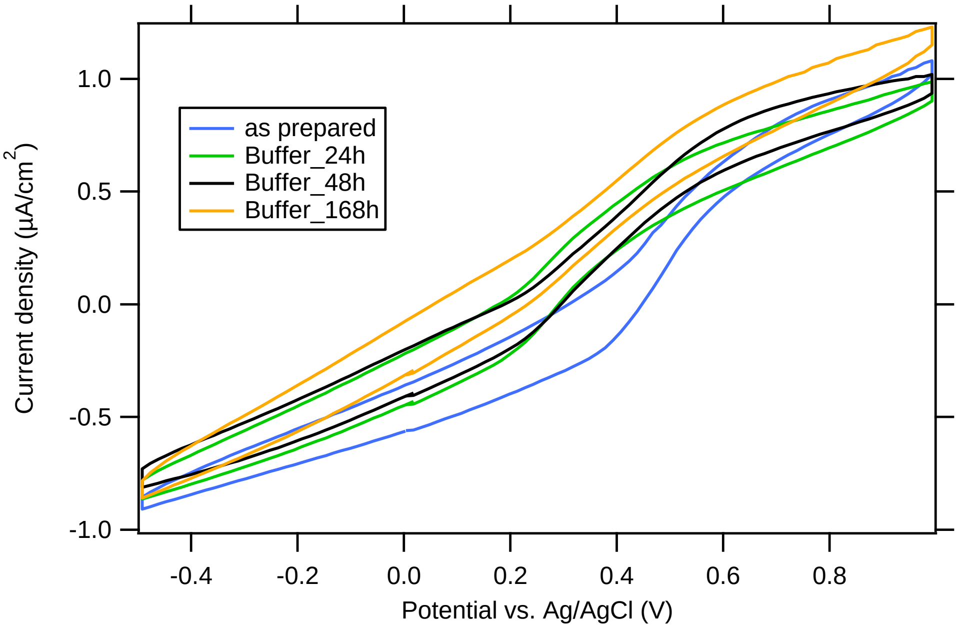

Figure S5: Details of the blocking properties of 17 nm Al_2O_3 films before and after exposure to phosphate buffer (pH 7.2) for 24, 48, and 168 hours. Cycling voltammetry in the presence of 0.5 mM $K_3[Fe(CN)_6]$ and 0.5 mM $K_4[Fe(CN)_6]$ in 0.5 M KCl. The scan rate is 50 mV/s.

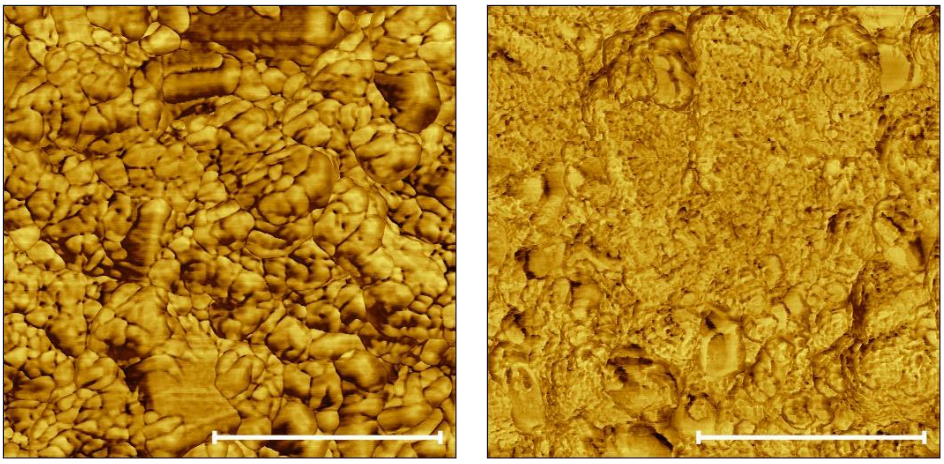

Figure S6: AFM phase image (1 µm × 1 µm) of an FTO substrate before (left) and after (right) electrochemical treatment (−1.2 V, 5 h, phosphate buffer pH 7.2). White bars represent 500 nm.

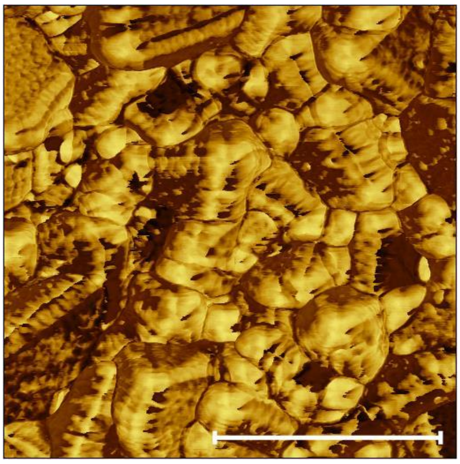

Figure S7: AFM phase image (1 µm × 1 µm) of FTO substrate with 10 nm Al_2O_3 film after electrochemical treatment (−1.2 V, 5 h, phosphate buffer, pH 7.2). The white bar represents 500 nm.

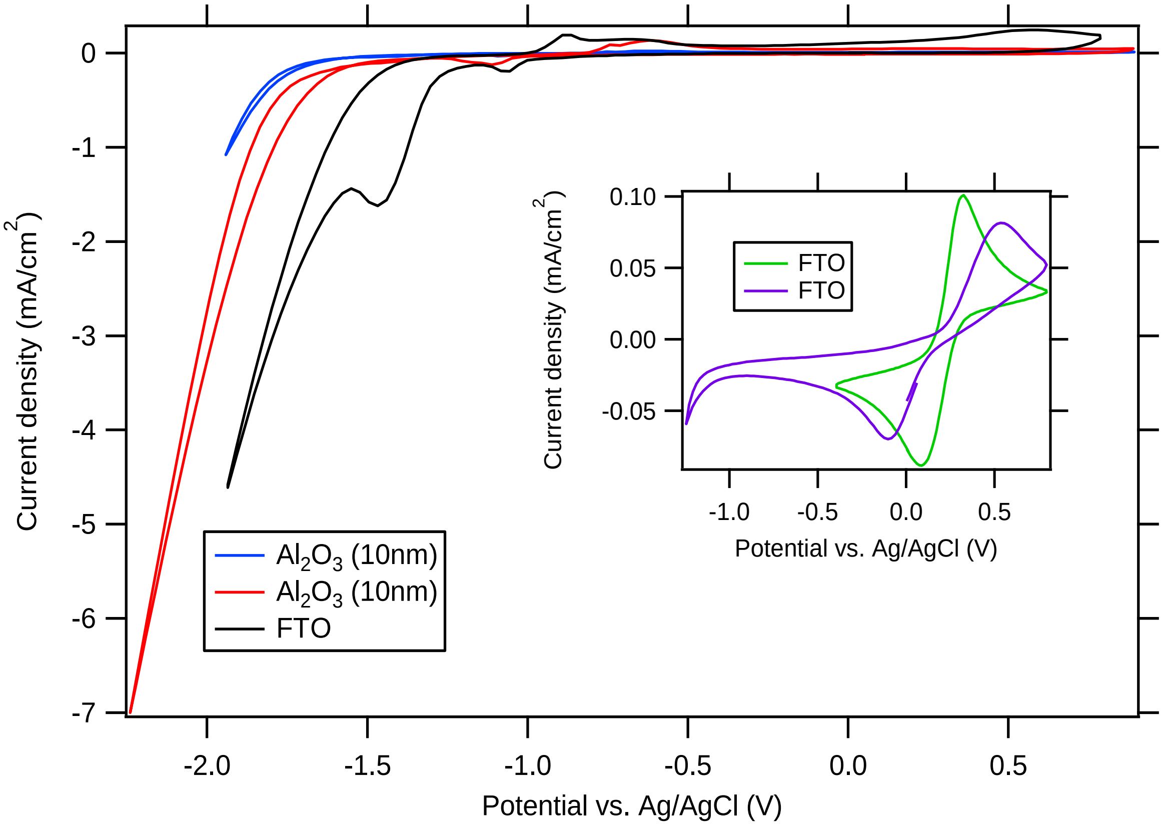

Figure S8: Cyclic voltammograms of 0.5 mM $K_3[Fe(CN)_6]$ and 0.5 mM $K_4[Fe(CN)_6]$ in phosphate buffer solution (pH 7.2) demonstrating a cathodic breakdown of FTO and its stabilization by coating with a 10 nm Al_2O_3 film. Inset: details of voltammograms on blank FTO with two different cathodic vertex potentials. The scan rate is 50 mV/s.

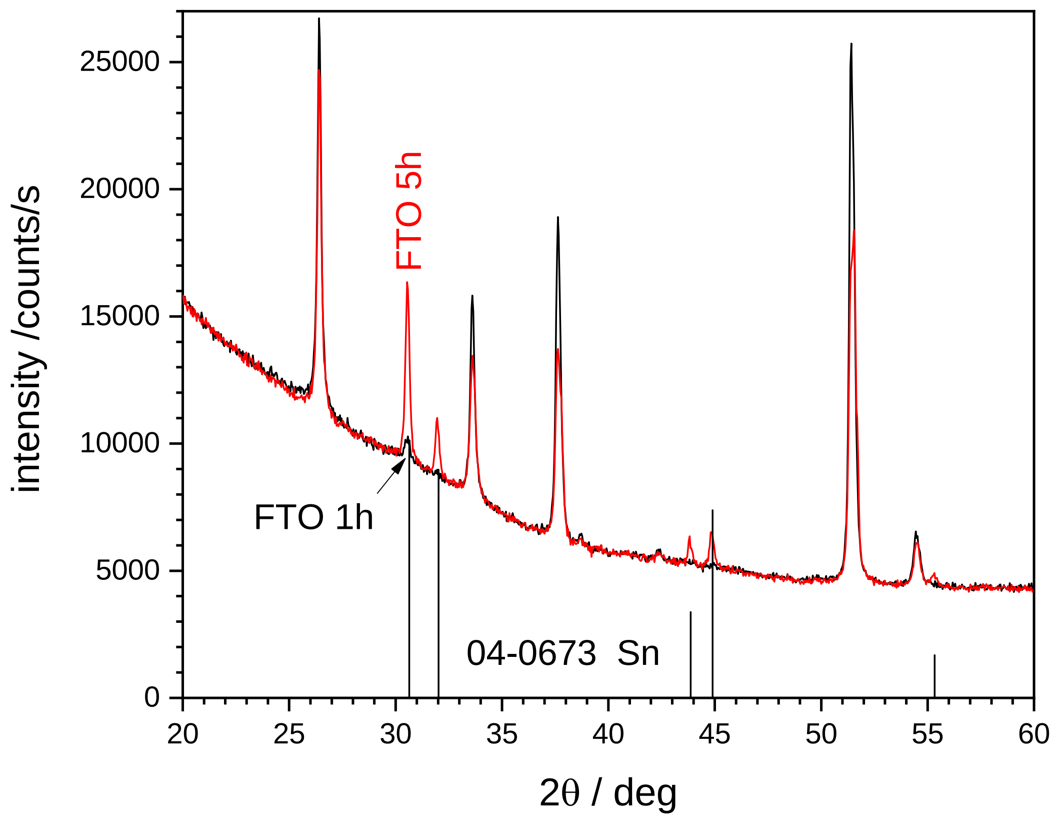

Figure S9: XRD patterns of unprotected FTO layers polarized at −1.2 V vs Ag/AgCl in buffer (pH 7.2) for 1 and 5 h. The Sn pattern was obtained from [3].

References

1. Kavan, L.; Tétreault, N.; Moehl, T.; Grätzel, M. *J. Phys. Chem. C* **2014,** *118,* 16408–16418. doi:10.1021/jp4103614

2. Krýsová, H.; Krýsa, J.; Kavan, L. *Beilstein J. Nanotechnol.* **2018,** *9,* 1135–1145. doi:10.3762/bjnano.9.105

3. Powder Diffraction File Alphabetic PDF-2 Data Base, International Center of Diffraction Data, Newtown Square, PA, USA, 1994.
